# Beyond Wet-to-Dry: A Rational Approach to Treating Chronic Wounds

**Published:** 2009-04-13

**Authors:** Johnson C. Lee, Swetha Kandula, Noëlle S. Sherber

**Affiliations:** ^a^ Johns Hopkins University School of Medicine, Baltimore, MD; ^b^ Johns Hopkins Wound Center, Baltimore, MD; ^c^ Johns Hopkins Department of Dermatology, Baltimore, MD

## Abstract

**Objective:** This article reviews the current recommendations for the classification and treatment of chronic wounds. With a rational approach and a thorough understanding of available treatment options, plastic surgeons can provide better-quality and more cost-effective wound care. **Methods:** The authors reviewed the literature on the history of wound care and on recent advancements in wound care and also summarized the current clinical practices of the Johns Hopkins Wound Center. **Results:** n/a. **Conclusions:** Optimized wound dressings decrease pain, diminish morbidity, and improve healing times.

Wound care is often performed in a manner that is anecdotal, inconsistent, and lacking in evidence base. In 2004, the direct costs of wound care in the United States exceeded 15 billion dollars.^1^ Wet-to-dry and gauze dressings remain the most widely used primary dressing material in the United States.^2^ While the newer-generation wound dressings are more costly than gauze, they decrease overall costs since they are more effective.^3^,^4^

## EVOLUTION OF WOUND CARE

While Sumerian cuneiform tablets depicted poultices being applied to wounds 4000 years ago, the ancient Egyptians advanced this practice by creating dressings composed of vegetable fibers, grease, and honey to provide absorbency, barrier protection, and antimicrobial activity, respectively. Around 150 AD, the Greek physician Galen published the observation that wounds from gladiatorial combat healed optimally in a continuously moist environment.^5^,^6^

Recent advancements in technology and in the understanding of human physiology have led to the commercial development of dressings that offer material improvements on these same ancient fundamental principles. Even so, gauze still remains the mainstay of wound care. We now know that patients with chronic wounds often have multiple underlying medical comorbidities, and the rate of wound healing is affected by multiple factors (Table 1). A chronic wound is defined as a break in the skin of more than 6 weeks' duration and are often difficult and time-consuming to treat.^7^ Wet-to-dry dressings are suboptimal, as they can delay wound healing by removing migrating epithelium and further cause pain by exposing sensitive nerve fibers in the wound bed.^8^

**Figure 1:**
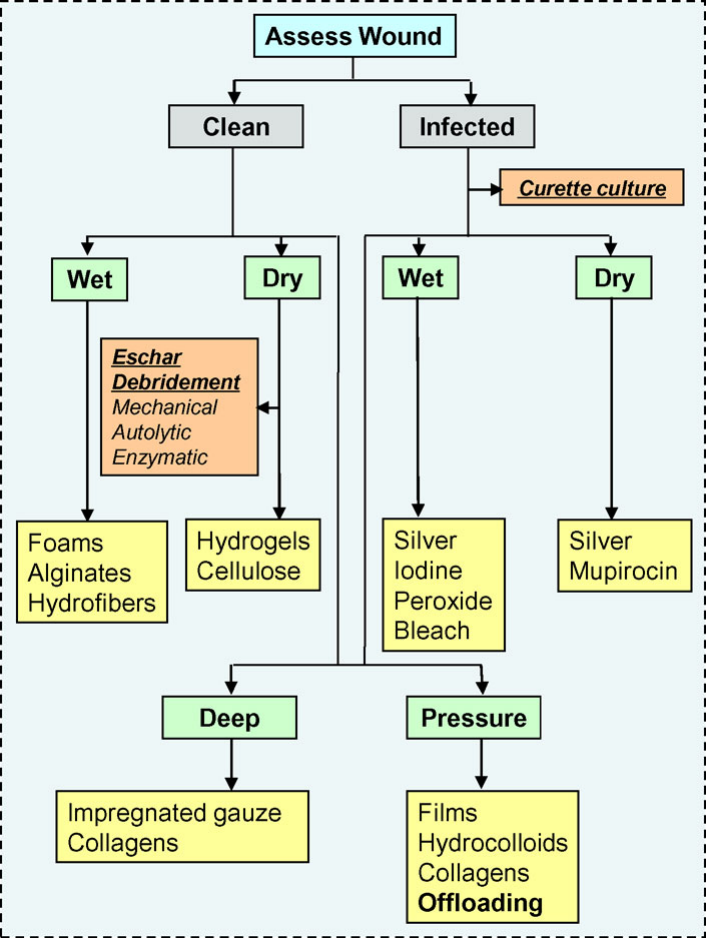
An algorithmic approach to wound dressing selection.

Today's plastic surgeons are heavily involved in caring for patients with chronic wounds, and, as such, are positioned to advance the quality of care. Assessing a wound's key characteristics enables a physician to select the optimal wound dressing for each patient (Fig 1).

## CATEGORIES OF WOUND DRESSINGS

### Occlusive dressings

Occlusive dressings create a moist wound healing environment, promote autolytic debridement, stimulate collagen synthesis and angiogenesis, and accelerate reepithelialization. Since they do not adhere to the wound bed, they reduce wound pain.

### Foams

Foams are best for wounds with mild to moderate exudate and provide thermal insulation. Polyvinyl alcohol foam impregnated with Methylene Blue and Gentian Violet also provides broad-spectrum bacteriostatic protection against microbes including methicillin-resistant *Staphylococcus aureus* (MRSA), vancomycin-resistant *Enterococcus* (VRE), and *Pseudomonas*.^9^–^11^ Foam has also been found to be preferable for pain reduction, patient satisfaction, and nursing time when compared with gauze dressings.^12^ Foam dressings should be changed every 3 days or sooner if needed on the basis of the amount of wound drainage. Do not use foams under compression as they produce ridges in the skin that can lead to breakdown.

### Alginates and hydrofibers

Alginates and hydrofibers are best for moderate to heavy exudate and have hemostatic properties. Alginates are composed of varying amounts of mannuronic and guluronic acids, the latter of which increases viscosity and allows the dressing to be removed as a single piece.^13^ These generally should be cut to fit the wound shape to prevent periwound maceration. These dressings should be changed every 3 days or sooner if needed on the basis of the amount of wound drainage. Do not use mannuronic acid–based alginates or hydrofibers for packing tunneled wounds as they form a gelatinous mass that can be difficult to retrieve completely.

### Hydrogels and cellulose

Hydrogels and cellulose donate moisture to wounds and are therefore used for dry and painful wounds. Sheet hydrogels help in transmitting nerve impulses across a wound, decreasing the perception of pain. Amorphous hydrogels can fill in cavities in larger wounds while maintaining a moist environment. When compared with gauze, hydrogels have been shown to be superior for debridement of diabetic foot ulcers.^14^ Hydrogels and cellulose dressings should be changed daily. A secondary dressing—such as petrolatum-impregnated gauze—is required to maintain moisture.

### Films and hydrocolloids

Films and hydrocolloids maintain a moist environment and protect the skin from friction and shearing forces. These can be left in place for up to 7 days. Skin injury can be prevented by lifting a corner of the dressing and stretching it parallel to the skin to break the adhesive bonds prior to removal.

## Antimicrobials

Wound healing is impaired by bacterial colonization, and a bacterial load of more than 100 000 colony-forming units per gram of tissue is considered to be deleterious. Curette culture has been proven to be the most accurate method for sampling infected wounds. Frankel et al^15^ have demonstrated that using a single brisk harvest with a 3-mm curette at the advancing border of the wound reliably and reproducibly produces 20 mg of tissue, an amount that is sufficient to perform quantitative cultures. Deep tissue biopsy remains the “gold standard” despite the fact that this is an invasive approach. Superficial skin swabs are considered inadequate because they do not provide information on the bacteria invading deep tissues. It has been demonstrated that anaerobic yield was higher for curette or deep tissue biopsy than for superficial swab although the facultative and aerobic populations demonstrate more similarity.^16^ When topical antimicrobials fail to heal a wound, systemic antibiotics are warranted on the basis of microorganism sensitivities. In addition, radiological evaluation is indicated for persistently infected wounds to investigate for underlying osteomyelitis.

### Silver

In the 1920s, the US Food and Drug Administration accepted colloidal silver as a wound treatment.^17^ Silver has broad-spectrum antimicrobial activity, even against MRSA and VRE but may be less effective against *Pseudomonas*.^18^ Silver products may contain sulfa, which is contraindicated in patients with known sulfa allergy. Silver dressings should be changed daily. Silver ions—not atoms—produce the clinical effect, so contact with fluid is necessary.

### Iodine

Iodine has excellent broad-spectrum antimicrobial activity but should be used with caution in patients with thyroid disease and in pregnancy. In particular, topical application of cadexomer iodine has been found to be effective for healing venous leg ulcers.^19^ Iodine dressings should be changed every other day. Concentrations of ≥ 1% iodine are toxic to skin, so cadexomer 0.9% iodine should be used for controlled release.

### Peroxide and bleach

Peroxide and bleach are commonly used antiseptics. Antiseptics are chemical agents that are broadly toxic to microbes and often inhibit wound healing. By contrast, antibiotics are narrow-spectrum agents with specific microbial targets. Peroxide and bleach-containing dressings should be changed daily. Bleach soaks should be performed using 0.025% concentration.

### Mupirocin

Mupirocin has good gram-positive coverage and is active against MRSA. Bacitracin does not provide coverage against MRSA. Mupirocin should be applied daily with dressing changes. Antibiotic ointments should be used sparingly, as wound and periwound skin has an increased incidence of developing contact dermatitis.^20^

## Debridement

Debridement converts chronic wounds to acute wounds and recruits neutrophils and macrophages that produce growth factors that promote wound healing. Mechanical debridement consists of wet-to-dry dressings, whirlpool baths, mist therapy, and surgical debridement. Autolytic debridement is achieved by most of the occlusive dressings. Enzymatic debridement can be achieved through the use of collagenase, papain, and urea. Collagenase is derived from fermentation by *Clostridium histolyticum* and has the ability to digest the collagen in the necrotic tissue. Urea is a protein denaturant and releases an active form of papain when they are used in combination.

## Impregnated gauze

### Hypertonic saline–impregnated gauze

Hypertonic saline–impregnated gauze provides absorption, debridement, and protection against microbial proliferation. It is appropriate for moist, sloughing, or hypergranulated wounds. It should be changed daily. Moistening the hypertonic saline–impregnated gauze prior to application decreases the pain that is associated with its use.

### Petrolatum-impregnated gauze

Petrolatum-impregnated gauze is used as a secondary dressing to keep primary dressings moist. It should be changed every other day. Some forms of petrolatum-impregnated gauze have antimicrobial ingredients, and these can be used as primary dressings.

### Polyhexamethylene biguanide–impregnated gauze

Polyhexamethylene biguanide–impregnated gauze has broad activity against MRSA and is suitable for packing deep wounds.^21^ This dressing should be changed twice daily. A concentration of 0.2% is needed for optimal efficacy.

## Collagens

Collagens are available from bovine, porcine, and human cadaveric sources. They can bind proteases and free radicals that are impediments to wound healing.^22^ An injectable form can be used to treat recalcitrant sinus tracts. Topical collagen dressings should be changed every 3 days. Collagens are especially useful to promote granulation tissue in protein-deficient or older patients.

## CONCLUSION

Not all chronic wounds are the same and therefore cannot be treated as such. Recent advances in wound dressing technology combined with improved understanding of wound healing physiology have made it possible to customize wound care. With a rational approach and a thorough understanding of available treatment options, plastic surgeons can provide better-quality and more cost-effective wound care. Optimized wound dressings decrease pain, diminish morbidity, and improve healing times.

## Acknowledgments

The authors thank Gerald Lazarus, MD, for reviewing this manuscript, and Yelena Frankel, MD, MPH, Margaret Fonder, MD, and the Johns Hopkins Wound Center.

## Figures and Tables

**Table 1:** Ideal wound environment

Moist
Free from exudate
Warm
Protected from trauma
Acidic
Protected from infection
Free from necrotic tissue
